# A Discourse on Brains

**Published:** 1907-08-15

**Authors:** C. M. Wright

**Affiliations:** Cincinnati, O.


### A DISCOURSE ON BRAINS.

BY PROF. C. M. WRIGHT, CINCINNATI, O.

Address to the graduating Class of 1907 of the Ohio College of Dental Surgery, May 9th, 1907.

When we say a business must be carried on by Brains, or a practice must be conducted with Brains—what do we mean?

What do we mean by "Brains." In a recent book on "Brains and Personality," the author W. Hanna Thompson, asks this question.

Is the Brain ot man an Eolian Harp or a Violin.

An Eolian Harp gives up its music to every breeze that happens to sweep across its strings without method and without will or distinction; while a Violin is played upon by an Intelligence, or Will, or Personality.

As I read the book, memory brought to mind the picture of a grand old ruin of a castle, that stands on a hill, a little way out trom Baden-Baden. The beauty of the situation—the views of the fine surrounding country and the interesting drive through the valley and up the mountain, makes a visit to the ruin, one ot the attractions of this popular German resort.

Across a casement in a fragment of a vine covered wall, somebody has strung a few fine wires and harp strings, and when the wind plays hide and seek and rushes through that gaping window, it sets these strings vibrating and the sweetest music is watted through the old ruin. Lovers sit about in nooks, waiting tor the music of the Eolian Harp, for it harmonizes well, with the thrills in their own hearts.

Elderly people stop to listen—and wonder if, in this weird vestige ot a past age, the spirits ot those who once lived there, still hover about, or wether it is the music ot the pipes of Pan that they hear.

The Eolian Harp responds to chance outside influences, just as the lower centres of man's nervous system does to animal instinct.

If the zephyrs play gently the tunes of the Harp are dulcet and low. If the wind blows boisterously, the vibrations of the strings clang and clash—and discord reigns.

Now let us turn to the Violin.

I have seen in a shop window a violin that could be bought for $1.25, and I once handled an instrument that cost $6,000.

Both were violins.

They looked alike to me.

They had the same number of strings—the same shape and size, and neither of them gave out a sound, to tell me that one was cheap, and that the other was a rare and valuable instrument.

They had another thing in common, and that was, that each required a player, a violinist—something with a Will—to make it respond as music. The wood and ebony and catgut were fashioned to bring out music, only at the will of a violinist. Then I thought. The trained hand and brain of a virtuoso can make even the $1.25 fiddle respond sweetly, while the untrained hand, when it tries to play on the $6,000 instrument, can call in the police.

The tool is not everything, a hand guided by a Personality must be behind the tool, and the Personality must be cultivated to effect the best results.

The Brain of Man is a combination of Violin and Eolian Harp.

Now if I may be allowed I should like to consider each member of this class a fiddle. You have stood a good deal from me for three years, and have been wonderfully polite and good-natured.

I'm sure you will forgive this, especially, when I add, that I should like also to compare all mankind, each one in this great audience—each learned professor on this platform and each weary and patient demonstrator to a violin. I want to insist too, that I am glad to be a fiddle and not altogether a mere Eolian Harp.

And I'll try to tell you why.

Anatomists and Physiologists have, from their dissections and studies of nerves and brains, for many years leaned toward the theory that we are not, *Free Agents*, but that we are governed in our higher, as well as in our lower brain activities—in our intellectual, as well as in our merely physical organization by impulses, such as hunger, anger, jealousy, self-preservation and love—just as is an Eolian Harp by the wind.

That these influences when mild and good, make us respond in mild and good ways—and when they are fierce and bad—we respond in fierce and bad ways.

When zephyrs play upon us we give out sweet music, and are amiable friends and good citizens, but when other environments, like strong appetites and temptations assail us we respond mechanically and naturally in a discordant way and are bad men, liars, or thieves perhaps. This theory of the Physiologist is quite generally accepted for we pray daily: "Lead us not into temptation."

The underlying doctrine is the same, i. e. that our inherited brain structure, or in other words—the kind of strings that make up our Eolian Harp Brains—and the kind of influence that play upon us, are entirely responsible for the kind of music we make in our lives.

Anatomists and Physiologists have experimented upon the brains and nerves of thousands upon thousands of frogs and pigeons, guinea pigs, dogs and monkeys, and they have been compelled to regard these nervous systems as only wonderfully built-up reflex machines. In insects and worms the machine is very simple and easily comprehended. In the higher animals which seem to show almost human intelligence, the reflex machine is extremely complex, and yet with all the complexity, the principle of action is the same.

Nerve matter responds to irritation, and every movement and action of a worm, or of a man is the result of nerve irritation, and effected by nerve impulses.

Everything that a man thinks or does, is the result of reflex action or the response to irritations through a machine. This machine is his brain and the nerves connected with it —and the brain and its nerves, is like an Eolian Harp and its strings.

This is true—as far as it goes—but it doesn't go quite far enough. It is without question a law of all brain and nerve action.

Irritate a nice quiet sedate gentleman's brain with a sufficient number of High-balls and he will respond "all right" "all right." He'll stagger in his walk, and look silly, and sing, and hiccough, and laugh, and shout and love you to death, and go right home and whip his wife.

All depending upon the spring in his brain which happens to be touched. You push the button and he does all the rest. He is a machine sure enough—a reflex machine.

But there is more in it than this.

There is a tract of brain substance in man, that can play the part of a violin. A Personality or Will has the power to take possession of certain parts of the brain, and mould and build those parts into intimate connection and association with the rest of the machine, so that they can by this same law of reflex action, control and regulate the higher and more complicated portions of the machine. These tracts have lately been definitely located and studied. We humans then have something that other animals have not.

Now, what is that something which is a gift to humanity alone?

It is this: Humans have the power to appropriate certain parts of their brains and to store these parts with *words* and *ideas* and set them off as the residence of a Personality or Will.

This *Will* acts as the director of the orchestra—or, rather as the first violinist in each of our brains.

This will-power is possessed by man alone—of all the animals—man is the only speaking creature.

Monkeys have *Speech Centres* in their brains, just like the speech centres in man's brain, but they can not possibly learn to use them. To talk is the privilege of man and of woman of course.

The Anatomist cannot by the most carefully scrutiny discover any very marked difference between a man's brain and the brain of a Chimpanzee. But the physiologist can show clearly, that certain little knots of brain matter in the brains of monkeys are never used and can't be brought into use. While man is capable by will-power of gradually developing these same kind of knots in his own brain and using them for the highest purposes—namely for seeing and hearing and remembering and speaking words, and for the storage, little by little, of ideas and thoughts.

Because of this ability man occupies the highest plane of responsibility and accountability. Mere machines are not responsible. These empty places are found in the brain of the child, but they are taken possession of very early in life, and occupied by a Personality and furnished little by little and kept in order by the growing Personality or Will according to its tastes and means.

Some Wills are allowed to remain weak and sluggish, and such furnish these rooms, which they have leased, with the basest and commonest necessities of life.

Others, add books to the empty cases, and pictures to the walls, and refined accessories of culture to the rooms. They make additions year by year of language, of general knowledge, of art, of history, of science and of moral and religious culture.

Remember, none of these things were there in the beginning, these were not furnished. Every one must furnish his own. The task begins at childhood and goes on through school and college days, and continues throughout life, day by day, and little by little; this is man's responsibility. This is the Responsibility which we as Human Beings cannot escape.

Notwithstanding the fact, that the law of reflexes is fundamental, and that this law allies us to all creatures, from the worm to the most highly developed Chimpanzee. We are placed upon a plane of the highest alps, while all other creatures dwell at the sea level.

Man is the only responsible animal, and he is responsible because he is a Man.

He is responsible to God, and to the laws of God.

He is responsible to Man—and to human laws—the laws of society and the laws of the state. He is responsible to himself, for he alone has the power of building by his own efforts at self-education, a personality, that controles and regulates his thoughts and conduct.

Capacity and quality has very little to do with this responsibility. If you only possess a $1.25 fiddle—study and practice with fingers and bow—over and over and over again with high ideals and a deep appreciation of your responsibility, and you will make that little fiddle sing like a little angel in heaven.

If you have a $6,000 Stradivarius and only strut about and boast that this valuable instrument belongs to you alone, and you leave it in its case for fools to gaze at, your personality amounts to nothing, for being a man, you are responsible for the music that you could bring out of your fine instrument.

May I close by urging each one of this class of 1907 to continue the work of furnishing his Brains, and to add day by day some small object to these select rooms in his Brain. A good book on this shelf, a fine sentiment on that, a bit of drapery here, an ornament there and a scrap of knowledge every day, until a splendid character is stamped upon everything that his self-educated Personality has furnished. This is worth while.

